# Surgical Management of Advanced Mandibular Osteonecrosis Utilizing a Contemporary Mandibular Reconstruction Plate in Patients Unsuitable for Free Flap Reconstruction—Preliminary Study and Case Series

**DOI:** 10.3390/jcm15051694

**Published:** 2026-02-24

**Authors:** Marios Fouzas, Evagelos Kalfarentzos, Kamil Nelke, Christos Perisanidis

**Affiliations:** 1 Oral and Maxillofacial Surgery University Clinic, Dental School, National and Kapodistrian University of Athens, Evangelismos General Hospital, 10676 Athens, Greece; gnath@evaggelismos-hosp.gr; 2 Maxillo-Facial Surgery Ward, EMC Hospital, Pilczycka 144, 54-144 Wrocław, Poland

**Keywords:** mandibular osteonecrosis, segmental mandibulectomy, plating system, mandible reconstruction, MRONJ, ORN, bridging plate

## Abstract

**Introduction:** Stage three osteonecrosis of the jaw (ONJ), whether medication-related (MRONJ) or osteoradionecrosis (ORN), often necessitates aggressive surgical management due to extensive necrosis, infection, and risk of pathologic fracture. While free flap reconstruction remains the gold standard post-segmental mandibulectomy, it may not be feasible for elderly or systemically compromised patients. **Objective:** The presentation of our own experience with advanced mandibular ONJ on patients managed exclusively with a contemporary titanium reconstruction plate system and to evaluate the clinical outcomes of this approach in the context of the current literature. **Methods:** From a group of 21 patients treated for ONJ, just four patients with Stage 3 MRONJ or Grade III ORN, unfit for microvascular surgery, underwent segmental mandibulectomy followed by alloplastic reconstruction using standard titanium plating. Outcomes were assessed clinically and radiographically over a follow-up period ranging from 3 to 20 months. A focused literature review was conducted to contextualize results. **Results**: All patients demonstrated stable reconstruction without plate exposure, fracture, or intraoral bone exposure during follow-up. Esthetic and functional outcomes are reported. No hardware complications were reported. The review of the literature supports plate-only reconstruction as a valid alternative for patients unsuitable for free flap surgery, especially when using rigid, anatomically adaptive systems with robust soft tissue coverage. **Conclusions**: Titanium plate–only reconstruction following segmental mandibulectomy can provide reliable short- to mid-term outcomes in selected patients with advanced ONJ. Used titanium plating systems appears to be a promising option.

## 1. Introduction

Osteonecrosis of the jaw (ONJ) is an adverse condition characterized by non-healing exposed bone in the maxillofacial region. Two major etiologies are recognized: medication-related ONJ (MRONJ) and osteoradionecrosis (ORN). MRONJ arises in patients exposed to antiresorptive or antiangiogenic agents (most commonly bisphosphonates or denosumab), without a history of head and neck radiation [1]. ORN, by contrast, is caused by radiation-induced ischemia of the jawbone, typically developing months to years after radiotherapy for head and neck cancer. Both conditions can progress to advanced stages with persistent bone exposure, infection, pain, and potentially pathologic fractures or fistula formation. The mandible is affected more often than the maxilla in both MRONJ and ORN [2,3]. Severity is often graded by clinical staging systems. In MRONJ, Stage 3 denotes exposed (or probed) necrotic bone with infection and complications such as an extraoral fistula, pathologic fracture, or osteolysis extending to the inferior border of the mandible or sinus floor [1,2,3,4]. ORN likewise has “severe” classifications (e.g., Notani Grade III) involving full-thickness necrosis or fracture. Advanced (Stage 3–Grade III) ONJ represents a challenging scenario that usually mandates surgical intervention to remove necrotic bone and prevent further morbidity [4,5,6,7]. It is also important that in order to minimize the possibility of local wound dehiscence, possible sources of inflammation in the area, including dentition, sinuses or others, should be firstly controlled. Therefore, patients should undergo total dental, laryngological and oral surgeon consults prior to surgery [8,9,10,11,12].

Osteonecrosis treatment varies based on the stage. Stage 1 usually responds to conservative therapy, with local sources of inflammation control and improvement of oral hygiene. Stage 2 treatment also includes pain control, antibiotics and surgical debridement wherever infection is present or suspected [12,13,14,15,16,17]. Stage 3 ONJ dictates more aggressive surgical approaches, including large resections and reconstruction using free vascularized flaps, as well as alloplastic reconstruction [6,7,8,9,10]. In our opinion, this alternative approach with the use of this newer plate system in patients with mandibular Stage 3 ONJ should be presented and discussed.

Although MRONJ and ORN differ in underlying pathology, they were discussed together in this study due to shared end-stage reconstructive challenges. No stratified, comparative, or equivalence conclusions between MRONJ and ORN are intended, and all observations should be interpreted descriptively.

The authors of this paper would like to present an alternative approach to mandibular reconstruction using only the recently introduced titanium bridging plate system proposed by Medartis (Basel, Switzerland). This plate system was used because of its unique design, which is different in geometry and properties from other reconstruction plates we had at our disposal. Our preliminary report indicates that in compromised patients who require surgery but are not eligible for microvascular reconstruction, titanium-only reconstruction provides a good alternative.

## 2. Materials and Methods

**Figure 1:**
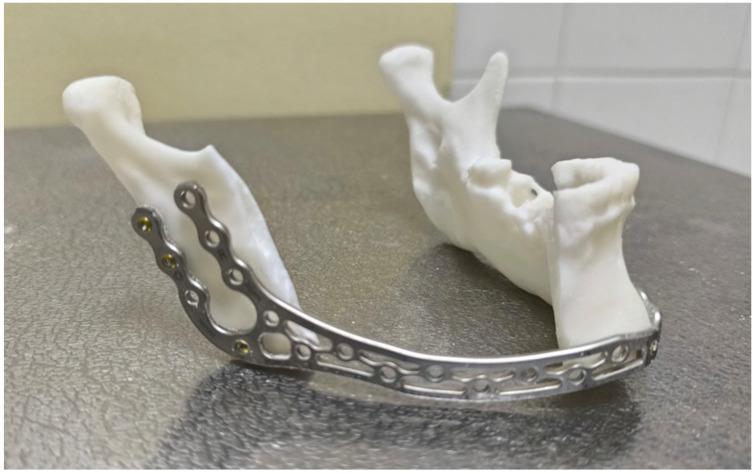
Medartis (Basel, Switzerland) MODUS^®^ 2 Mandible bridging plate on a 3D-printed mandible model.

For this case series, we present four cases of patients with Stage 3 MRONJ or Grade III ORN of the mandible that were deemed unfit for free flap surgery and were thus treated with segmental mandibulectomy and alloplastic reconstruction of the mandible, utilizing only the MODUS^®^ 2 Mandible Trilock 2.0 bridging plates by Medartis (Figure 1). For our patients, we used 3D-printed mandible 1:1 ratio models we printed in-house and did plate pre-bending on them to save time during the operation. We used load-bearing bridging plates, utilizing all 3 available designs (with 17, 22 and 26 holes), fixed with bicortical 2 mm and 2.3 mm titanium screws, both locking and non-locking. We also modified plates by removing parts (holes), where their design dictated possible. The pre-bent plates were then sterilized in this shape. During surgery, after adequate mandibular bone exposure and final assessment of the mandibulectomy extent, plates were checked for their anatomical fit and small corrections were made if necessary. A few screws were placed to ensure stable position, the plates were removed and mandibulectomy was performed. Plates were then re-positioned using the same holes, to ensure their identical position. Small adjustments were again made if necessary before final plate fixation. Authors will present and discuss outcomes from our proposed plating protocol (PPP).

**Figure 2:**
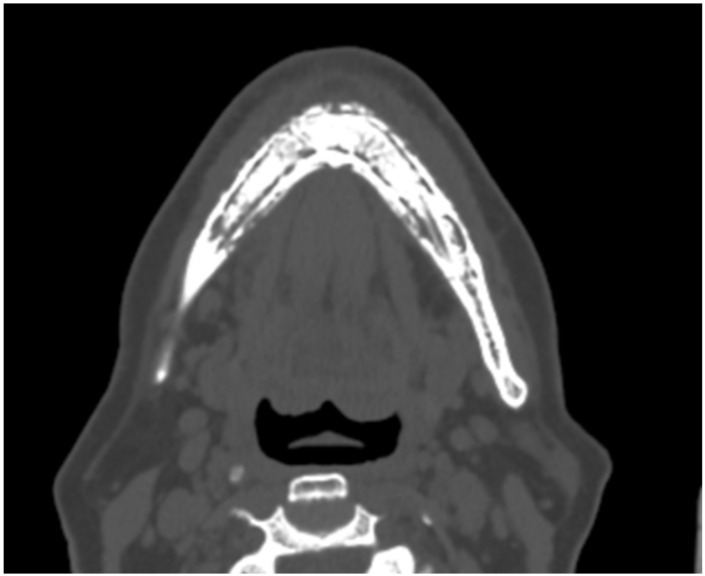
CT scan of one of our patients showing typical radiographic findings of Stage III MRONJ (full-thickness bone necrosis, diffuse periosteal reaction and sequestrum formation).

In our studied patient group the most common clinical symptoms and radiological signs related with each ONJ case included pain, edema, purulent fistulas intraorally and extraorally, exposed bone, abscess formation and radiological findings like periosteal reaction, loss of normal bone architecture, sequestrum formation and pathologic fractures (Figure 2).

In this article we used the classification system proposed by the AAOMS in their 2022 position paper on MRONJ, which proposes a 4 stage system (0, 1, 2 and 3) [1,2,3]. Stage 3 dictates exposed necrotic bone with evidence of infection and one or more of the following: necrotic bone extending beyond the alveolar bone, pathologic fractures, extraoral fistulas, oroantral/oronasal communication and osteolysis up to the inferior border of the mandible or the sinus floor [1]. For mandibular ORN classification, we used the Notani grading method, which is most commonly used in the literature. Here, Grade III represents ORN that extends below the inferior alveolar nerve canal, the presence of skin fistulas or pathologic fractures [13].

Out of twenty-one patients that we examined with MRONJ and ORN in our department in the last two years, four were unfit for surgery because of very poor general condition and prognosis, twelve were treated with simpler procedures, such as sequestrectomy or local debridement, and one younger patient was programmed to receive a fibula free flap for mandibular segmental mandibulectomy, following full-thickness mandibular MRONJ. Four patients were finally selected to receive a segmental mandibulectomy and reconstruction, using only the new MODUS^®^ 2 Mandible Trilock bridging plates in our PPP approach.

Our inclusion criteria for this case series were the presence of advanced osteonecrosis (Stage 3 MRONJ or Notani Grade III ORN) of the mandible and an overall patient health condition that made them unfit for free flap surgery, such as coronary disease, diabetes and poor prognosis due to metastatic bone disease, but otherwise fit for general anesthesia. Patients’ own choice for a shorter surgical procedure was also taken into account.

Exclusion criteria included the presence of lower stage osteonecrosis that could be treated with less invasive surgical procedures, younger and healthy patients that could be treated with free flap reconstruction, and too frail patients with extremely poor prognosis that were assigned palliative care.

The goals for the use of our plating protocol included improvements in our patients’ general quality of life (QOL), their ability to eat, the eradication of microbial infections in the area, relief from pain and other symptoms, and esthetic restoration. Due to a lack of an official QOL questionnaire, we based our assessment on simple yes or no questions we asked the patients at the follow-up appointments, regarding whether they are satisfied with their ability to masticate and to speak, their maximum mouth opening, the presence of pain in the operated area or their temporomandibular joint, and their outside appearance. The patients were instructed to apply rigorous oral hygiene measures and to immediately report any signs of inflammation, pain, purulence, trismus or irregular mobility in the mandibular area.

For our targeted review of the literature, we searched for scientific articles in the PubMed, Google Scholar and ScienceDirect directories, focusing mostly on the last ten years of data (2015–2025) and restricted to English articles. The literature search started by including the AAOMS position paper on MRONJ as a reference point for definition and staging of the disease, as well as Notani’s widely used article for mandibular ORN staging [1,13]. Then, the search focused on identifying publications in which MRONJ, ORN, segmental mandibulectomy and reconstruction were addressed in combination. Search terms used were different combinations of MRONJ, ORN, Stage III–3, reconstruction, free flap, titanium plate, mandibulectomy, and compromised patients. Exclusion criteria were the absence of reference to reconstructive strategies and the focus on strictly non-surgical measures, early-stage ONJ or ONJ of the maxilla.

Titles and abstracts were manually screened for relevance, followed by full text assessment of potentially eligible articles. Since this article is a preliminary report and case series, and was not prospectively designed as a systematic review, early-stage screening counts were not recorded, and searches were not scanned for duplicates. Approximate numbers are therefore provided to enhance transparency. About 50 articles were assessed in full text form for eligibility. Of these, only 14 were included, with 12 referring to segmental mandibulectomy as a treatment and only 2 to reconstruction with only a titanium plate.

Another search about the Medartis plate system, using terms such as Medartis, Modus 2 ^®^, Mandible, and Trilock, was done, and after abstract and title screening 3 studies were assessed in full text and included in our article. Other studies were excluded due to them not referring to segmental mandibulectomy or to the specific plate system studied.

The citation list was completed with articles based on authors’ own knowledge, as well as editors’ and reviewers’ recommendations.

## 3. Results

A total of four patients chosen for this series were all treated with segmental mandibulectomy and reconstruction using only a titanium bridging plate by Medartis in the authors’ PPP approach. Details about each patient are provided in the following section (Table 1). We note that these patients were referred to our hospital from other hospitals, carrying oncologists’ notes regarding their health status and prognosis. They were not discussed in a multidisciplinary tumor board.

**Figure 3:**
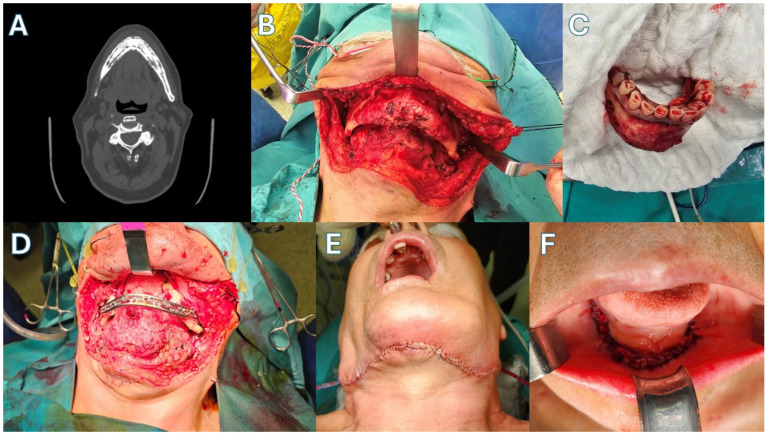
Case 1. (**A**) Pre-op CT scan. (**B**) Necrotic mandible segment. (**C**) The surgical necrotic specimen after excision. (**D**) Bridging of the mandibular defect. (**E**) Extraoral incision closure. (**F**) Intraoral incision closure.

**Figure 4:**
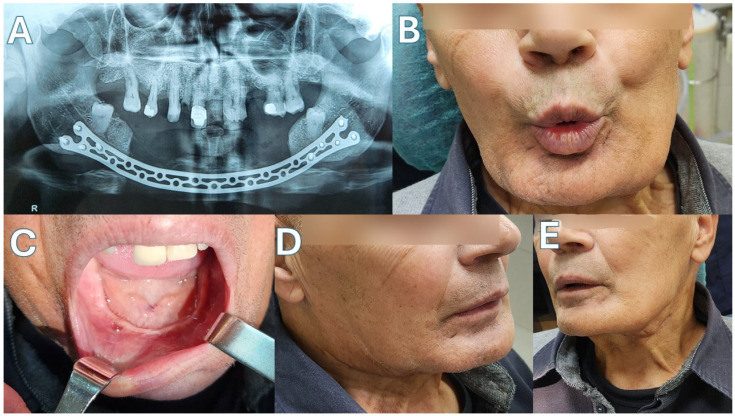
Case 1. (**A**) Post-op panoramic X-ray. (**B**) The patient 5 months after the operation, showing good marginal mandibular nerve function. (**C**) Intraoral view. (**D**) Right mandibular contour. (**E**) Left mandibular contour.

Patient 1 (Figure 3 and Figure 4)—Our first case is a 69-year-old male with a history of metastatic prostate carcinoma. The patient had received years of denosumab therapy and presented with edema, pain and extraoral fistulas in the area of the mandibular symphysis, giving the diagnosis of Stage 3 MRONJ. Treatment began with oral doxycycline for 3 months. This led to an improvement in his clinical condition, with the resolving of the fistulas and the marked reduction in the edema. The CT scan after this period showed full-thickness mandibular lesions, with sequestrum formation and periosteal reaction. Given the patient’s diagnosis and his health status, a segmental mandibulectomy of the necrotic bone and a reconstruction using only a titanium plate of the new Medartis ^®^ MODUS^®^ 2 Mandible Trilock 2.0 bridging plate was decided, as shown in the pictures. Great care was taken to preserve the soft tissues around the plate, in order to prevent future plate exposure. The patient made an uneventful recovery, presenting with no local inflammation, no necrosis spread and good esthetics with a satisfactory mandibular contour. Unfortunately, the patient succumbed to his metastatic disease 14 months later.

**Figure 5:**
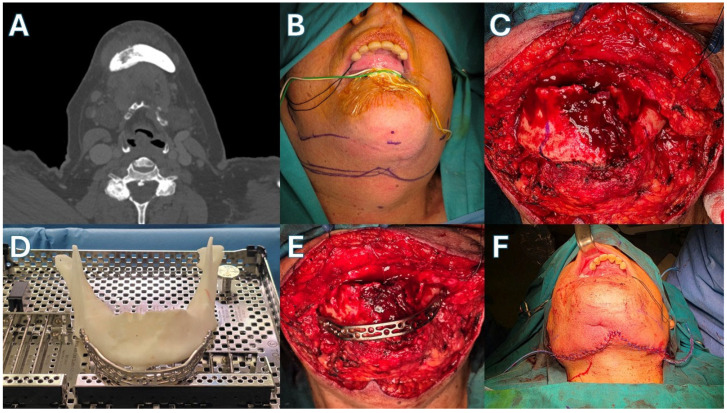
Case 2. (**A**) Pre-op CT showing necrotic mandibular lesion. (**B**) Incision design and marginal mandibular nerve neuromonitoring cables in place. (**C**) Exposed mandible with lesion. (**D**) Pre-bended titanium plate using a 3D model. (**E**) The plate in place. (**F**) Final incision closure.

**Figure 6:**
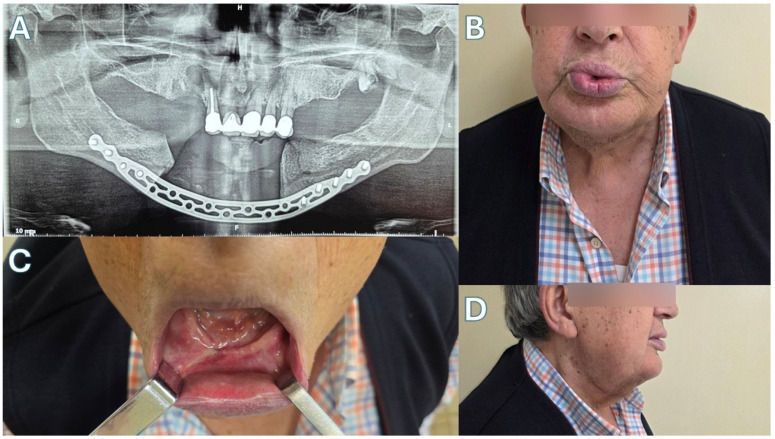
Case 2. (**A**) Post-op panoramic X-ray. (**B**–**D**) Extraoral and intraoral views of the patient, 1 month post-op.

Patient 2 (Figure 5 and Figure 6)—A second, 67-year-old patient, with a history of metastatic prostate carcinoma under denosumab therapy for 1 year, presented in the outpatients’ office with swelling and pain of the mental mandibular area. After a full examination and work-up, the diagnosis of a submental abscess due to Stage 3 MRONJ was made. The abscess was drained in the office and the patient was placed on oral amoxicillin–zclavulanate and metronidazole antibiotic treatment for 10 days. Inflammation and pain subsided. Surgical treatment of MRONJ was warranted. Because of this overall health condition, we treated the patient with a segmental mandibulectomy and titanium plate-only reconstruction. The preservation of the soft tissues and the careful coverage of the titanium plate with the musculature of the area are observed in the pictures below. The patient, 3 months after surgery, has no symptoms or signs of recurrence and is happy with his esthetic outcome.

**Figure 7:**
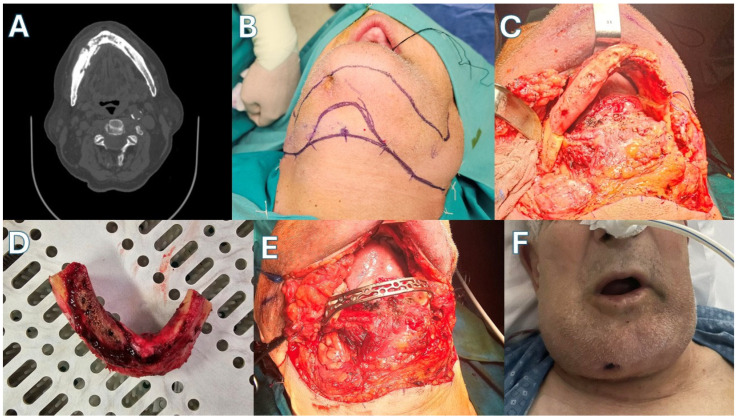
Case 3. (**A**) CT showing necrotic lesions. (**B**) Incision design. (**C**) Necrotic mandible exposed. (**D**) Resected surgical specimen. (**E**) Defect bridging. (**F**) The patient immediately post-op.

**Figure 8:**
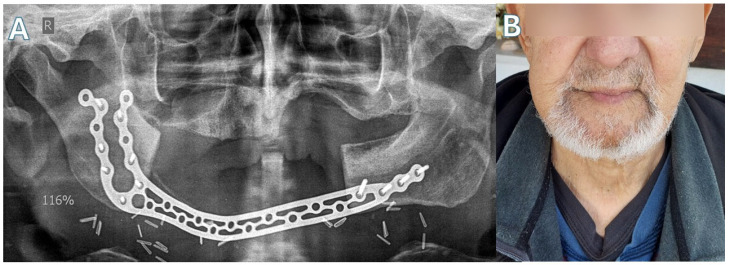
Case 3. (**A**) Post-op panoramic X-ray. (**B**) The patient 20 months after surgery.

Patient 3 (Figure 7 and Figure 8)—Another male patient with a history of coronary disease, type II diabetes and intravenous 5-year zolendronic acid and 1-year denosumab therapy, due to metastatic prostate carcinoma, was admitted to the hospital because of mandibular swelling and discomfort. The patient also reported an extraoral fistula in the right submandibular area that had improved with oral antibiotic treatment. CT showed full-thickness mandibular necrotic lesions that extended approximately from the right ramus area to the symphysis. The patient was operated on with a segmental mandibulectomy of the right mandible and reconstruction with a titanium bridging plate, as the first two patients. The resection in this case was extensive, so plate bridging was more challenging. As with other patients, a new Medartis MODUS^®^ 2 Mandible Trilock 2.0 plate was used. Twenty months after surgery, his metastatic disease has progressed with new bone metastases, and the patient has difficulty in mobilization. However, the surgical outcome of the mandible is stable, with no plate exposure, no on-site inflammatory lesions, no intraoral exposed bone and a satisfactory facial contour, according to the patient’s testimony.

**Figure 9:**
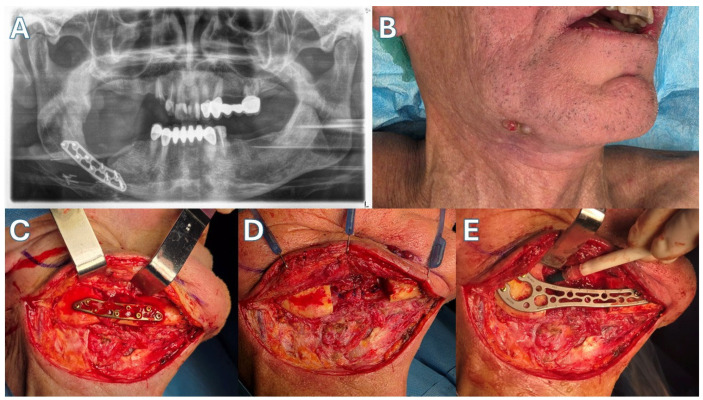
Case 4. (**A**) Pre-op panoramic x-ray, showing the old Trilock^®^ Trauma plate and the pathologic mandibular fracture. (**B**) Extraoral fistula visible pre-op. (**C**) The fracture and necrotic lesion exposed. (**D**) After mandibular resection. (**E**) New bridging plate in place.

**Figure 10:**
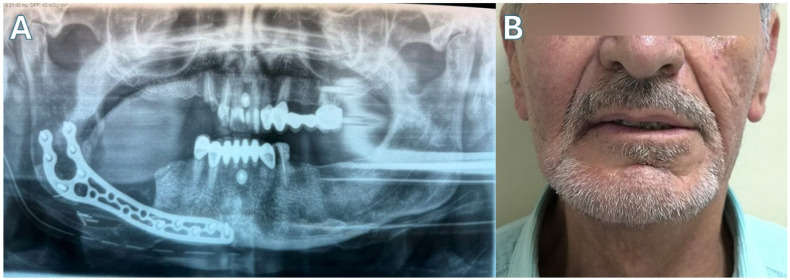
Case 4. (**A**) Post-op X-ray. (**B**) The patient 3 months after surgery.

Patient 4 (Figure 9 and Figure 10)—The fourth and final patient of this case series is a 70-year-old male that has undergone head and neck radiotherapy for metastatic cervical lymphadenopathy of unknown primary 3 years ago. The patient had presented with exposed bone intraorally and pain in the right mandibular region, giving the diagnosis of osteoradionecrosis (ORN). He had been operated on 6 months ago with a marginal mandibulectomy of the area, which led to an intraoperative mandibular fracture and was restored with a Medartis^®^ MODUS 2 Mandible Trauma plate, as shown. However, 5 months after this procedure, the patient relapsed, presenting with intraoral and extraoral purulent fistulas, intense pain of the site and localized edema. The CT scan and panoramic X-ray showed the spread of necrotic lesions at the site of the previous surgery, as well as a pathologic mandibular fracture. This time, a segmental mandibulectomy was indicated. The patient was informed of the possibility of free flap reconstruction, although his irradiated neck would make finding adequate vessels for anastomoses difficult and the surgery relatively risky. He chose to undergo the simpler procedure that is described. Intraoral and extraoral fistulas and necrotic bone were completely excised. A titanium-only bridging reconstruction strategy was utilized. The patient recovered well and 3 months after surgery no recurrence is observed, and he is satisfied with his functional and esthetic results. The patient remains cancer-free and in good general health.

In total, the goals of our PPP were met. No patient presented with osteonecrosis recurrence, plate exposure or plate fracture in our series. Patients reported an improvement in their overall QOL, staying pain-free during our observation period, with satisfactory esthetic results (based on patients’ own testimony), no inflammation of the area and no perceived irregular mandibular mobility. Ability of speech, mastication and TMJ function was maintained, according to patients’ own experience. No nerve deficits were noted, significant scarring was not present and other bacterial contamination was not present in the observation period. The authors acknowledge that our number of patients is small and the time of follow-up observation brief. Therefore, this article remains a descriptive case series and its hypothesis-validating ability remains inherently limited. However, it is apparent that the reconstruction of segmental defects using only the new Medartis MODUS^®^ Mandible 2 Trilock bridging plates has some promising results that need to be further evaluated with larger patient groups and longer follow-up periods. Our results are summarized in the following Table 2.

## 4. Discussion

Advanced ONJ cases often require aggressive surgical management. For maxillary defects, maxillectomy can be performed, and many reconstruction options are available, such as primary closure, local flaps (like FAMM, tongue flap, submental flaps), or free flap reconstruction with or without other combinations of approaches [13,18,19,20]. Another effective use of adjacent tissues for bone coverage and reconstruction was presented by Zhou et al., where authors used ipsilateral submandibular gland translocation with reconstruction plates with overall good success rates [7]. Surgical treatment of ONJ must not only secure the bone position and reduce possible pathologic fracture occurrence, but also excise any fistulas, decrease bacterial infection, remove all granulation tissue, and improve patients’ well-being. If such bone lesions with purulent discharge and necrotic bone are not treated, some patients might develop skin inflammation or abscesses, especially if the patient is immunocompromised or has other present co-morbidities. In severe maxillofacial conditions such as these, systemic inflammatory markers have been shown to reflect disease burden and response to treatment, highlighting the importance of timely infection control and comprehensive surgical management [21]. Improved oral hygiene, wound care, as well as skin care in ONJ can greatly affect patients’ long-term outcomes [22].

For the mandible, segmental mandibulectomy is regarded as the gold standard for Stage 3 ONJ. Evidence shows significantly improved outcomes following complete resection of the necrotic bone compared to conservative or partial surgical treatments. [6,14]. In MRONJ, Otsuru et al. report complete healing in 92.3% of cases treated with segmental mandibulectomy, particularly when performed with adequate margins [14]. For ORN, resection of devitalized bone followed by reconstruction with vascularized free flaps also yields high success rates [2]. Mitate et al. emphasize the role of any reconstruction to promote stable bone position, improve mastication, maintain good esthetics and oral cavity balance, stating that those results can be more predictable and accurate, when using patient-specific planning and solutions [23].

Reconstruction of mandibular defects post-mandibulectomy is traditionally accomplished with vascularized bone flaps, such as fibula osteocutaneous flaps, which restore continuity and function and allow for future dental rehabilitation [10,13]. Flap success rates exceed 90% in experienced centers, even among elderly or co-morbid patients [7,11]. Out of the free flaps used, the scapula and the iliac crest flaps are described, but for most authors the fibula free flap has been the preferred choice, because of bone and skin paddle size versatility. The ability to direct the vascular pedicle to the contralateral side is also important, specifically in cases of previous neck irradiation [2,7]. However, free flap surgery is complex and not feasible for all patients due to prolonged operative time, the need for microvascular expertise, and donor site morbidity.

As an alternative, some surgeons use titanium reconstruction plates to bridge the defect without a bone graft. This approach is especially relevant for frail patients or those with poor prognosis [7,8]. For osteoradionecrosis, Huang et al. emphasize that the role of microsurgery remains a treatment of choice, but free flap reconstruction can also have important complications and patients unfit for those surgeries should be treated with other alternatives [24]. Plate-only reconstructions can achieve good short-term outcomes, with primary healing ranging 60–85% across series [7]. Complications such as plate exposure or fracture are mitigated by using rigid, CAD-/CAM-designed plates and ensuring robust soft tissue coverage [8]. In some cases, spontaneous bone regeneration has been observed, particularly when periosteum is preserved [9]. Many studies have been made regarding the specific plate design for bridging these gaps. A finite element analysis study by Demir et al. highlights that biplanar custom reconstruction plates had more favorable stress distribution [25]. A Bräuer et al. study indicated that single laser-sintered CAD–CAM plates used in elderly, multi-morbid patients in palliative situations had better outcomes when compared to manually bent miniplates [9].

For patients with very poor prognoses, either oncologic or with serious co-morbidities, that make surgery under general anesthesia not feasible, pharmaceutical and other non-surgical treatments have also been studied in the literature. Chlorhexidine 0.12–2% rinsing, hyperbaric oxygen therapy, antibiotics, PRP and other therapies have been used, demonstrating various results [26,27]. The most prominent pharmacological treatment for MRONJ and ORN has been the supplementation with pentoxifylline and tocopherol, also known as PENTO protocol, or PENTOCLO protocol, where clodronate is also added. This regimen, which appears to systemically promote blood circulation, has shown promising results for patients, with reported complete healing for ORN up to 100% and an improvement in symptoms and healing, both clinical and radiographic, in cases of MRONJ [26,28]. Even in advanced disease, while surgery is generally the treatment of choice, there have been reports of Grade III ORN patients with pathologic fractures that achieved bone healing without surgery using only PENTO [29]. However, in cases such as some of our patients with active malignant disease under treatment, there have been studies showing that antioxidant supplementation can work against tumor control, increasing recurrence rates and reducing survival rates [30]. It is obvious that more studies are needed to fully understand this supplementation protocol. In our series of cases, since surgery under general anesthesia was possible, and there have been some indications of possible hazards of PENTO for oncologic patients, we decided to treat them with surgery alone.

Concerning the specific use of the MODUS^®^ 2 Mandible TriLock bridging plates we used, there are some limited data available about their outcomes when compared to other reconstruction plate systems. Peters et al. stated that compared to other mandibular plate systems reported in the literature, the Medartis MODUS^®^ Mandible Trauma/Reco 2.0–2.5 TriLock plate system showed improved clinical outcomes, with a plate exposure rate of 17.07% in the bridging plate group and only 4.17% in the mandibular plate group, and no cases of plate breakage in either group. Pseudarthrosis was observed in just 4.17% of cases using mandibular plates. These rates are notably lower than those associated with many other systems, some of which report exposure rates exceeding 20% and breakage rates up to 18% [31]. According to Gielisch et al., the Medartis MODUS^®^ 2 Mandible TriLock mandibular reconstruction system demonstrated a notably low complication rate, with no instances of plate fractures and only one case (5%) of plate exposure across 20 patients. This contrasts with previously reported exposure rates of 15–46% and plate fracture rates up to 16% for other reconstruction systems. Additionally, the system allowed for high primary stability even in challenging conditions, such as salvage surgery after osteonecrosis or tumor resection, due to its locking mechanism and optimized plate design [32]. Steyer et al., in their latest publication regarding the same plate system we used, report that the new Medartis MODUS^®^ 2 Trilock bridging plate system had fewer plate exposures (20% compared to 45%) and fewer on-site infections (0% compared to 30%) than the previous Medartis MODUS^®^ Reco 2.5 system, showing no difference in plate fractures, as none were observed in both groups. It is also mentioned that the new plate system was deemed by surgeons to be better in intraoperative handling, although the old system had greater bending capabilities, allowing it to be bent in all planes, due to its geometry [33].

Our limited experience in the use of the new MODUS^®^ 2 Mandible Trilock system has so far been good. There have been no patients with plate exposure, no unsatisfactory esthetic results, specifically concerning the mandibular contour, and no titanium plate fractures. The plates, showing noticeably less rigidity and probably more elasticity than the previous Medartis MODUS ^®^ Reco plates, can be more easily bended to mimic the anatomical structures they replace, resulting, in our case, in slightly shorter plate bending and surgery times. This difference in elasticity is probably the cause of the absence of plate fractures in the literature and in our own patients. However, the literature is still limited and more clinical and biomechanical studies are needed to assess our early observations. The only downside we can observe is the fairly large plate height (namely 17 mm), which can be a problem in cases of severely atrophic mandibles. When it comes to our patients’ own experience, the absence of complications and symptoms with the new plate system seems to impact their quality of life positively.

While free flap reconstruction offers superior functional outcomes and lower long-term complication rates, titanium plate-only reconstruction remains a valid option for patients unfit for major surgery. It offers a simpler, shorter procedure that resolves infection and maintains mandibular continuity. Surgeons must weigh benefits against potential risks such as hardware failure or an inability to support prosthetic rehabilitation [8]. In cases such as the patients of our series, presenting with metastatic bone disease, poor prognosis, co-morbidities and relatively old age, reconstruction of the mandible using only an appropriate reconstruction plate system can be the treatment of choice, providing satisfactory esthetic and functional results, while minimizing the risks. Surgery duration for our patients was 2–3 h. A fibula free flap surgery in our hospital would require around 8 h to perform. Patients such as ours benefit from this significantly shorter surgical time. It is also important to note that in most cases of ONJ, there is no shortage of soft tissues surrounding the bone defects, and therefore adequate soft tissue coverage of the plates is usually feasible without the need for osteocutaneous or osteomyocutaneous flaps. The aforementioned coverage of plates with the surrounding soft tissues is crucial for the prevention of intraoral or extraoral wound dehiscense. We therefore took care to maintain all soft tissues around the plates. The local musculature was sutured around the plates to ensure a first robust layer of soft tissue, and the oral mucosa was carefully sutured as a second layer, using simple or mattress sutures to ensure watertight closure. This technique is highly recommended by the authors in cases such as these. Bone edges were also smoothed using burs to prevent injury to the surrounding soft tissues. In cases with significant intraoral wounds, the patients were fed through a nasogastric tube for 2 weeks, to minimize dehiscence chances. Finally, prefabricated plates, like the ones we used, are generally low-cost compared to patient-specific implants or extensive free flap surgeries.

Limitations of this paper include the following: the small number of patients treated with the authors’ PPP approach, lack of long term-outcomes, necessity to improve the study with comparison with quality of life assessment scales and the effects of other pharmacological agents on healing and patients’ general condition. The nature of this paper remains one of a descriptive case series and its findings should only be interpreted as such.

## 5. Conclusions

Stage 3 MRONJ or Grade III ORN of the mandible present a challenging clinical scenario necessitating surgical intervention. Segmental mandibulectomy offers the highest chance of disease resolution when combined with appropriate reconstruction. Free flap reconstruction is the preferred method for fit patients, offering long-term durability and improved function. However, for patients who are not good candidates for microvascular surgery, titanium plate-only reconstruction is a viable alternative with acceptable outcomes. With advancements in plate technology and soft tissue coverage techniques, this method provides symptom relief and structural support, aligning with palliative or transitional goals in complex ONJ cases. The new Medartis MODUS^®^ 2 Mandible Trilock bridging plate system has some promising results, but the limited patient number and short follow-up periods in our cases series make it clear that our findings should be regarded as exploratory and hypothesis-generating only. Further studies with more patients and longer observation times are needed in order to provide a better image of the new system’s strengths and weaknesses.

## Figures and Tables

**Table 1:** Patient characteristics.

Case	Health History/Morbidity	Age/Sex	Resection Site	Surgery Indication
1	Metastatic prostate Ca. Denosumab therapy. Poor oncologic prognosis.	69/Male	Mental area	Pain/edema/extraoral fistula
2	Metastatic prostate Ca. Denosumab therapy. Coronary disease—MI history. Moderate oncologic prognosis.	67/Male	Mental area	Abscess formation/pain/local soft tissue inflammation
3	Metastatic prostate Ca. IV zolendronic acid and denosumab therapy. Coronary disease—type II diabetes. Poor oncologic prognosis.	68/Male	Lateral right mandible	Mandibular edema/discomfort
4	Neck irradiation history/metastatic lymph node disease/vessel-depleted neck. Previous ORN surgery in the area. ORN recurrence. Patient choice for shorter surgery.	64/Male	Lateral right mandible	Pathologic fracture/previous plating failure/pain and discomfort

Abbreviations: MI = Myocardial Infarction; IV = Intravenous; ORN = Osteoradionecrosis.

**Table 2:** Table of results based on our observations and patients’ reported yes or no answers.

Patient No.	Follow-Up	Pain	ONJ Recurrence	Mastication Satisfaction	Speech Satisfaction	TMJ Function Satisfaction	Esthetics Satisfaction	Plate Fracture/Exposure
1	14 months	No	No	Yes	Yes	Yes	Yes	No
2	3 months	No	No	Yes	Yes	Yes	Yes	No
3	20 months	No	No	Yes	Yes	Yes	Yes	No
4	3 months	No	No	Yes	Yes	Yes	Yes	No

Abbreviations: ONJ = Osteonecrosis of the jaw; TMJ = Tempormandibular joint.

## Data Availability

The datasets used and/or analyzed during the current study are available from the corresponding author upon reasonable request.
